# Identification of Host–Protein Interaction Network of Canine Parvovirus Capsid Protein VP2 in F81 Cells

**DOI:** 10.3390/microorganisms13010088

**Published:** 2025-01-05

**Authors:** Hongzhuan Zhou, Huanhuan Zhang, Xia Su, Fuzhou Xu, Bing Xiao, Jin Zhang, Qi Qi, Lulu Lin, Kaidi Cui, Qinqin Li, Songping Li, Bing Yang

**Affiliations:** 1 Beijing Key Laboratory for Prevention and Control of Infectious Diseases in Livestock and Poultry, In-Stitute of Animal Husbandry and Veterinary Medicine, Beijing Academy of Agriculture and Forestry Sciences, Beijing 100097, China; hanfu2002@sohu.com (H.Z.); zhh20qe@163.com (H.Z.); sxia2013@163.com (X.S.); fuzhouxu@163.com (F.X.); xiaobing1972@sohu.com (B.X.); 17710300295@163.com (J.Z.); q1258840198@163.com (Q.Q.); 18601147804@163.com (L.L.); ckd2024syphu@163.com (K.C.); qin99l02@163.com (Q.L.); 2 School of Life Science and Biopharmaceutics, Shenyang Pharmaceutical University, Shenyang 117004, China

**Keywords:** CPV, VP2, protein interaction network, FHL2

## Abstract

Canine Parvovirus (CPV) is a highly contagious virus that causes severe hemorrhagic enteritis and myocarditis, posing a major threat to the life and health of dogs. The molecular mechanism by which VP2, the major capsid protein of CPV, infects host cells and utilizes host cell proteins for self-replication remains poorly understood. In this study, 140 host proteins specifically binding to CPV VP2 protein were identified by immunoprecipitation combined with liquid chromatography–mass spectrometry (LC-MS/MS). Subsequently, the protein Interaction Network (PPI), the annotation of gene ontology (GO) and the database of Kyoto Encyclopedia of Genes and Genomes (KEGG) were constructed for in-depth analysis. The results showed that CPV VP2 protein participated mainly in cell metabolism, cell biosynthesis, protein folding and various signal transduction processes. According to the results of proteomics analysis, we randomly selected seven proteins for co-immunoprecipitation verification, and the experimental results were consistent with the LC-MS/MS data. In addition, our study found that the expression level of the VP2-interacting protein FHL2 mediated CPV replication. Preliminary studies have shown that knockdown of FHL2 promotes CPV replication by decreasing the expression of interferon β (IFN-β) and interferon-stimulated genes (ISGs), while overexpression of FHL2 can inhibit the replication of CPV by up-regulating the expression of IFN-β and related ISGs. This study lays the foundation for elucidating the potential function of CPV VP2 protein in the process of viral infection and proliferation which provides a theoretical basis for the design of antiviral agents and vaccines.

## 1. Introduction

Canine Parvovirus (CPV) causes an infectious disease and serious harm to dogs. It is highly contagious and has a high mortality rate, causing economic losses. The most common route of infection is the fecal–oral route or contact with contaminated surfaces. The virus first invades the pharynx, begins to replicate when it reaches the oropharynx, and spreads to other organs through the blood. It mainly attacks intestinal epithelial cells and cardiomyocytes, causing severe hemorrhagic enteritis and myocarditis [1]. It is most contagious 3-6 days after infection. Since the first outbreak in 1978, CPV has undergone an evolution from CPV-2a to CPV-2b to CPV-2c, with the emergence of new variants and an increasing trend in its distribution and infection rate [2].

Canine parvovirus (CPV-2) belongs to the genus Parvovirus of the family Parvoviridae, and contains a single-stranded negative linear DNA genome of approximately 5.12 kb [3,4,5]. The CPV-2 genome contains two open reading frames (ORFs), encoding two structural proteins VP1 and VP2 and two non-structural proteins NS1 and NS2 [6,7]. Non-structural protein 1 (NS1) of parvovirus (CPV) is a multifunctional protein with site-specific DNA binding, incisional enzyme, ATPase and helicase activity [8,9], which plays an important role in virus replication, cytotoxicity, DNA packaging and pathogenicity [10,11]. CPV structural protein 2 (VP2) is the main capsid protein, accounting for about 90% of the CPV-2 capsid, which determines tissue tropism, the antigenicity and host range of the virus [2]. In addition, the VP2 protein contains several important B cell epitopes in the N-terminal domain and the loop domain, which can induce effective neutralizing antibodies during CPV infection [12]. Therefore, VP2 protein is currently the first choice as a candidate antigen in the development of CPV genome subunit vaccines. This means that the CPV VP2 protein plays a crucial role in CPV–host cell interactions and can be used as a potential drug target to control CPV infection.

The process of virus infection of host cells is complex, multi-step, and often highly virus-specific [13]. Viruses must bind to and enter host cells and control cellular functions through host cell protein–pathogen protein interactions to complete their life cycle [14]. CPV VP2 is a structural protein, although there have been reports of host cell proteins interacting with CPV VP2 [15]. However, the extensive interaction spectrum of CPV VP2 and cellular proteins remains unknown. In addition, co-immunoprecipitation (Co-IP) combined with liquid chromatography–mass spectrometry (LC-MS/MS) technology is used to study the interaction between viruses and host proteins; however, it has not been used for comprehensive analysis of CPV VP2. In this study, we identified proteins interacting with CPV VP2 by immunoprecipitation (Co-IP) combined with liquid chromatography–mass spectrometry (LC-MS/MS), and analyzed by a protein interaction network diagram, GO and KEGG analysis, which laid a foundation for further understanding the pathogenic mechanism and immune escape mechanism of CPV.

## 2. Materials and Methods

### 2.1. Cells and Virus

The F81 cells (collected by the Cell Bank, Chinese Academy of Sciences) were grown in RPMI 1640 medium (Gibco) supplemented with 10% fetal bovine serum (Gibco), 100 U/mL penicillin, and 0.1 mg/mL streptomycin, and cultured at 37 °C, 5% CO_2_. HEK 293T cells and MDCK cells (collected by the American Type Culture Collection (ATCC))) were cultured in DMEM (Gibco) supplemented with 10% FBS (Gibco). The New CPV-2a strain SD6 (297Ala, 426Asn) was isolated and identified by our laboratory, and the accession number of its VP2 encoding gene in GenBank is MN101724.

### 2.2. Antibodies and Reagents

β-Actin monoclonal antibody (AC-15), goat anti-rabbit IgG (H+L) secondary antibody, and goat anti-mouse IgG (H+L) cross-adsorbed secondary antibody were purchased from Thermo Scientific (Thermo Scientifice, Waltham, MA, USA). The primary antibody against parvovirus (CPV1-2A1) was acquired from Santa Cruz Biotechnology (Santa Cruz Biotechnology, Dallas, TX, USA). The antibodies used for immunoprecipitation, ANTI-FLAG M2 (F1804) and Anti-c-Myc (M4439), were sourced from Sigma-Aldrich (Sigma-Aldrich, Saint Louis, MO, USA). Anti-GST (10000-O-AP) antibody used for GST pull-down was purchased from proteintech (Proteintech, Manchester, UK). Anti-c-MYC magnetic beads (HY-K0206) and Anti-FLAG magnetic beads (HY-K0207) used for immunoprecipitation were purchased from MedChemExpress (MedChemExpress, Monmouth Junction, NJ, USA). Additionally, T4 DNA ligase (M0202), and restriction enzymes XhoI (R0146), BamHI (R0136), EcoRI (R3101), KpnI (R3142), and SalI (R0138) were all procured from New England Biolabs (NEB, Ipswich, MA, USA).

### 2.3. Plasmid Construction and Cell Transfection

Primers were designed based on the full-length VP2 gene and its gene sequence (MN101724), and the full-length DNA fragment of CPV VP2 was amplified by polymerase chain reaction (PCR) and then cloned into the vector p3xFlag-CMV-14-VP2 (YouBio, Changsha, Hunan, China). The full-length cDNA sequences of fca FHL2 (gene ID: 101100121), fca CCT7 (gene ID: 101080777), fca CCT5 (gene ID: 100101123), fca LSG1 (gene ID: 101086415), fca WDR5 (gene ID: 101082447), fca DNAJB11 (gene ID: 101100077), and fca PRIM1 (gene ID: 101080554) were amplified from F81 cells using specific primers and cloned into vector pCMV-Myc-C (YouBio, Changsha, Hunan, China), respectively. fca CCT7 (gene ID: 101080777) and fca FHL2 (gene ID: 101100121) were cloned into vector pGEX-4T-1 (YouBio, Changsha, Hunan, China), respectively. The primers used are listed in Table 1. HEK293T or F81 cells were grown to 70% to 90% confluency on the plate before cell transfection. The indicated plasmids were added using Lipofectamine™ 3000 transfection reagent (Thermo Fisher Scientific, Waltham, MA, USA) according to the manufacturer’s protocol.

### 2.4. SDS-PAGE and Western Blotting

Cells were lysed using lysis reagent followed by the separation of proteins via sodium dodecyl sulfate polyacrylamide gel electrophoresis (SDS-PAGE). Subsequently, these proteins were transferred to polyvinylidene fluoride (PVDF) membranes (Millipore, Burlington, MA, USA). The membranes were closed with 5% Milk-TBS-Tween20 for 1 h at room temperature and washed three times with TBST. The membranes were incubated with primary antibodies overnight at 4 °C. After three to five washes with TBST, the membranes were incubated with the corresponding secondary antibodies for 1 h at 37 °C. Immunoreactive bands were detected with SuperSignal™ West Pico Plus Chemiluminescent Substrate Kit (Thermo Scientificial, Waltham, MA, USA) and images were obtained with Amersham Imager 600 (GE Healthcare, Pittsburgh, PA, USA).

### 2.5. Expression of Recombinant Proteins

The pGEX-4T-1, pGEX-4T-1-FHL2 and pGEX-4T1-CCY7 plasmids were transformed into *E.coli* BL21 (pLysS) cells. Single colonies were selected for amplification in LB medium at 37 °C and then induced overnight at 16 °C with 1 mM isopropyl-β-D-thiogalactoside (IPTG). GST, GST-FHL2 and GST-CCT7 proteins were incubated with Pierce glutathione agarose beads (21516; Thermo, Rockford, IL, USA) for 2.0 h at 4 °C. In accordance with the manufacturer’s guidelines, the proteins GST, GST-FHL2, and GST-CCT7 were individually eluted using an elution buffer that contained 2.0 mg/mL of reduced glutathione.

### 2.6. Co-Immunoprecipitation (Co-IP) and Glutathione S-Transferase (GST) Pull-Down Assays

For Co-IP, Plasmids were transfected in HEK 293T cells and, after 48 h, lysed with cell lysis buffer and centrifuged at 12,000× *g* for 15 min. The supernatant was treated with protein A/G plus agarose (Beyotime Biotchnology, Shanghai, China) for 2.0 h at 4 °C. After three washes with PBST, immunoprecipitation was performed using anti-Flag magnetic beads or anti-c-Myc magnetic beads (MedChemExpress, Monmouth Junction, NJ, USA). Subsequently, Western blotting was performed using anti-FLAG antibody (Sigma-Aldrich, Saint Louis, MO, USA) and anti-c-Myc antibody (Sigma-Aldrich, Saint Louis, MO, USA). The GST pull-down experiment was conducted following the guidelines provided by the manufacturer, using 3xFlag-VP2 as prey protein. GST, GST-CCT7 and GST-FHL2 proteins were immobilized on glutathione agarose beads and incubated with the prey proteins for 4.0 h at 4 °C. The proteins underwent SDS-PAGE and were examined through Western blotting with mouse monoclonal antibodies targeting GST or FLAG.

### 2.7. Liquid Chromatography–Mass Spectrometry (LC-MS/MS)

Coomassie blue-stained gels from Co-IP experiments were pooled and analyzed by LC-MS/MS for protein identification. Protein identification was performed by liquid chromatography–mass spectrometry (LC-MS/MS) analysis in APTBio (Shanghai, China). The steps are briefly described below. LC-MS/MS analysis was performed on a timsTOF Pro mass spectrometer (Bruker) coupled to NanoGlass. Peptides were loaded onto a C18 reversed-phase analytical column (homemade, 25 cm length, 75 μm inner diameter, 1.9 μm, C18) in buffer A (0.1% formic acid) and separated with a linear gradient of buffer B. The mass spectrometer was operated in positive ion mode. The mass spectrometer collected ion mobility MS spectra in the mass range of *m*/*z* 100-1700 and 1/k 0 of 0.75–1.35, followed by 10 cycles of PASEF MS/MS with a target intensity of 1.5k and a threshold of 2500. Active exclusion was enabled with a release time of 0.4 min. The MS raw data of each sample were merged and searched using MaxQuant software for identification and quantification. Max Missed Cleavages was set to 2, variable modifications was set to Oxidation(M), Acetyl(Protein N-term), and the enzyme was set to Trypin. Proteins found in the corresponding negative control samples were eliminated from the data set, to eliminate nonspecific binding interactions.

### 2.8. Construction and Analysis of Protein–Protein Interaction Network

The protein interaction network is composed of proteins interacting with each other, which participate in various links of life processes such as biological signal transmission, gene expression regulation, energy and substance metabolism and cell cycle regulation. Therefore, the mapping of interaction networks (PPIs) is very important for understanding cell organization, biological processes, and functions. In this study, we used the STRING database (https://cn.string-db.org/ (accessed on 27 October 2024)) and Cytoscape version 3.10.1, and used all the experimentally obtained data sets to generate the CPV VP2 host–protein interaction network. NCBI gene names were used to represent proteins throughout the study.

### 2.9. GO and KEGG Pathway Analyses

The host proteins that specifically bind to CPV VP2 were enriched for GO functional enrichment (http://geneontology.org (accessed on 27 October 2024)) or gene ontology (GO) analysis using Cytoscape software (version 3.10.1) and plug-in GOclue, and GO enrichment classification histograms were drawn to reflect the distribution of enrichment items in biological processes (BPs), cellular components (CCs), and molecular functions (MsF). (Supplementary Table S2). The KEGG database (http://www.genome.jp/kegg (accessed on 27 October 2024) and Cytoscape software (version 3.10.1) and plug-in GOclue were used to analyze the signal pathway of host protein enrichment that specifically binds to CPV VP2, and a histogram was drawn based on the enrichment results of the signal pathway (Supplementary Table S3). GO and KEGG pathways with *p* values < 0.05 were selected for significant enrichment.

### 2.10. RNA Interference

Interference sequences were designed and synthesized by GenePharma (GenePharma, Shanghai, China) based on the gene sequence of FHL2, as follows (Table 2). According to the manufacturer’s instructions, A concentration of 20 pmol si-FHL2 or negative-control si-NC was transfected into F81 or MDCK cells with Lipofectamine™ RNAiMAX (Thermo Fisher Scientific, Waltham, MA, USA) according to the manufacturer’s instructions, and then the subsequent experiments were carried out.

### 2.11. RNA Extraction and Quantitative PCR

In order to detect changes in the expression of interferon-related genes in cells, we infected cells with 0.1 MOI CPV in F81 cells with FHL2 knockdown or overexpression. Twenty-four hours after infecting the cells, poly (dA:dT) (0.5 μg/mL) (Invivogen, San Diego, CA, USA) was transfected with lipo3000 (Thermo Fisher Scientific, Waltham, MA, USA), and the cells were collected after 6h. Total RNA in the sample was extracted using RLT lysis buffe, and using FastKing RT kit (with gDNase) reverse-transcribed into cDNA. The reagents utilized for quantification included SuperReal PreMix Plus (SYBR Green) (TIANGEN, Beijing, China), and the reaction system was set up following the manufacturer’s guidelines. Amplification was carried out using the CFX Connect™ Real-time Fluorescence Quantitative PCR Detection System (Bio-Rad, Hercules, CA, USA). The relative expression of mRNA was calculated using β-actin as the internal reference gene, and the 2^−ΔΔCT^ method was used. The specific primers are shown in Table 3.

When detecting viral copy number, we infected cells with 0.1 MOI CPV for 48 h in F81 cells with FHL2 knockdown or overexpression, collected cells for three freeze–thaw cycles, and extracted viral DNA using the Quick DNA extraction kit (Cwbio, Suzhou, Jiangsu, China). According to the previously reported method, the quantitative standard curve was prepared using the pMD-VP2S positive plasmid stored in the laboratory, and the DNA copy number of the VP2 coding gene in the sample was determined using the generated standard curve [16]. The reaction system and instrumentation used for the samples were the same as above.

### 2.12. Statistical Analysis

All statistical analysis experiments used three repeated settings. Data were statistically analyzed using GraphPad Prism. Results of data analysis are shown as mean ± standard deviation (mean ± SD). Statistically significant differences were assessed using the Student *t*-test; * *p* < 0.05, ** *p* < 0.01, *** *p* < 0.005 and **** *p* < 0.001 were considered statistically significant.

## 3. Results

### 3.1. Identification of Host Proteins Interacting with CPV VP2 in F81 Cells by LC-MS/MS

**Figure 1:**
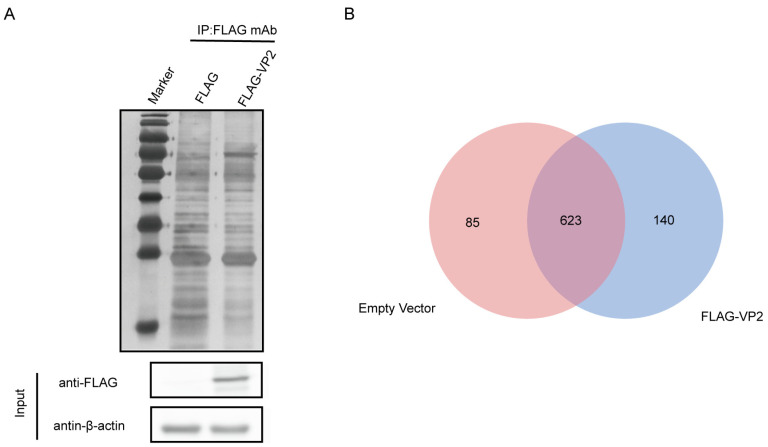
Identification of host proteins interacting with CPV VP2 protein. (**A**) The p3xFlag-CMV vector (2 μg) or p3xFlag-VP2 (2 μg) was transfected into F81 cells. Immunoprecipitation assay was performed 24h after transfection and analyzed on SDS-PAGE followed by silver staining. Lane 1, protein molecular weight ladder; lane 2, empty p3xFlag-CMV control; lane 3, p3xFlag-VP2 transfection. (**B**) Wayne plots with host proteins bound to CPV VP2 from empty p3xFlag-CMV control and p3xFlag-VP2, respectively. Proteins are indicated by their respective NCBI gene names (Supplementary Table S1).

To investigate the role of CPV–host interaction in CPV proliferation, we used co-immunoprecipitation (Co-IP) combined with liquid chromatography–mass spectrometry (LC-MS/MS) to identify host proteins that interact with VP2. The p3xFlag-VP2 plasmid and empty FLAG were transfected into F81 cells. Samples were collected 24 h after transfection. After sample lysis, co-immunoprecipitation experiments were performed using magnetic beads coupled to FLAG antibodies. The eluate was subjected to SDS-PAGE electrophoresis and silver staining was performed to visualize the host proteins that bind to CPV VP2 (Figure 1A). LC-MS/MS identified the interaction between CPV VP2and F81 cell proteome. Sequencing results showed that a total of 763 host-cell candidate proteins that bind to CPV VP2 were identified in cells transfected with p3xFlag-VP2. Among them, 623 cellular proteins were also present in the lysate of F81 cells transfected with the p3xFlag-CMV empty vector, and the remaining 140 (3 of these proteins do not have NCBI gene names) host proteins specifically interacted with CPV VP2, which can be further analyzed (Figure 1B).

### 3.2. Construction of Protein–Protein Interaction Network

**Figure 2:**
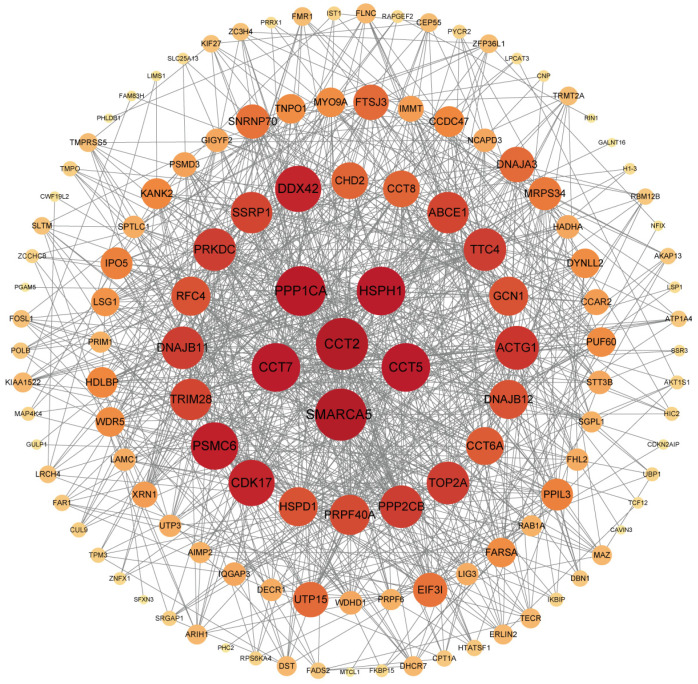
Construction and analysis of protein–protein interaction network. The STRING database and Cytoscape3.10.1 were used to construct and draw the interaction network diagram of host proteins interacting with CPV VP2 protein. The node is the host protein, and the edge represents the interaction between the nodes. The higher the degree, the larger the node, and the darker the color. Proteins use their respective NCBI gene names.

In vivo, proteins usually play a series of biological roles through complex interactions. Protein–protein interaction (PPI) is very important for understanding cell organization, biological processes and functions, and is a decisive aspect of molecular biology. Here, we used the STRING database to draw the interaction network between host proteins bound by CPV VP2 protein for network structure and function analysis (Figure 2). In the PPI analysis, it was observed that the network density (0.348) and the number of edges of the network (872) were significantly higher than the expected number of edges (675), indicating that the number of interactions was higher than expected. In the PPI network diagram, protein folding-related proteins CCT2, CCT5, CCT7, and heat shock protein HSPH1 are located at the center of the network, followed by proteins such as PPP1CA, DNAJB11, DNAJB12 and CCT8, etc.

### 3.3. GO Analysis

**Figure 3:**
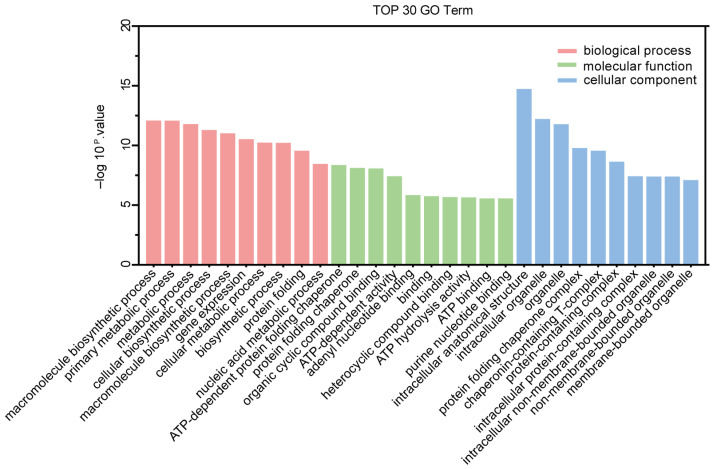
Gene ontology (GO) analysis based on the interaction between cellular proteins and CPV VP2. GO analysis was performed using the Gene Ontology Resource website. Protein GO distributions were categorized into three classes: biological processes (BP), molecular function (MF) and cellular component (CC). The detailed GO terms are shown in Supplementary Table S2.

To identify the functions of CPV VP2–host protein interactions, we annotated the proteins that specifically interacted with CPV VP2 with gene ontology, and classified them into three categories: biological process, molecular function, and cellular component through enrichment analysis (Figure 3, Supplementary Figure S1). Many biological processes including protein folding, macromolecule biosynthetic process, and metabolic process were affected. In addition, cellular components such as protein folding chaperone complex, chaperonin-containing T-complex and non-membrane-bounded organelle were mainly accommodated. Adenyl ribonucleotide binding, ATP-dependent protein folding chaperone and ATP binding are enriched in the molecular function category.

### 3.4. KEGG Analysis

**Figure 4:**
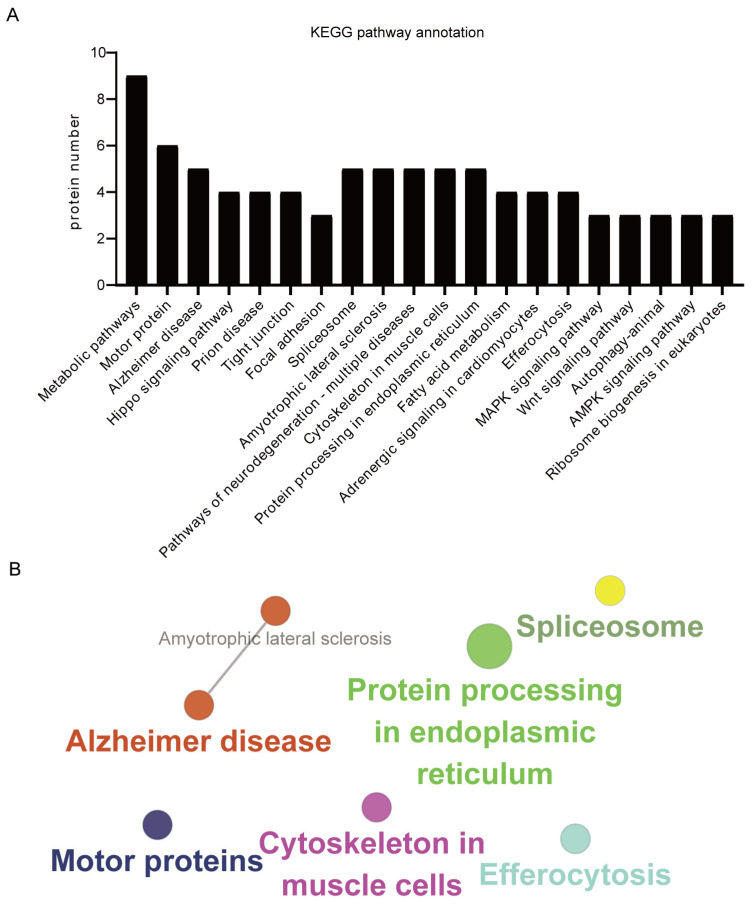
KEGG pathway enrichment analysis. (**A**) Enrichment pathway analysis of Canine Parvovirus (CPV) VP2 interacting proteins, using the Kyoto Encyclopedia of Genes and Genomes (KEGG) functional annotation pathway database (Supplementary Table S3). (**B**) CPV VP2 protein-interacting proteins analyzed by KEGG functional annotation using the GOclue plug-in in Cytoscape software, *p* < 0.05.

To further understand the proteins in CPV VP2 that interact with the host, we performed KEGG pathway enrichment, which is used to understand the advanced functions and utility of biological systems from genomic and molecular information. We selected the top 20 most representative enrichment pathways (Figure 4A,B). The results show that most proteins are involved in the processes of Ribosome biogenesis in eukaryotes, protein processing in endoplasmic reticulum and autophagy in animals. They also have a significant impact on signaling pathways, including AMPK signaling pathway, Wnt signaling pathway and Hippo signaling pathway, etc. Furthermore, these proteins may play key roles in regulating different processes, such as protein processing, fatty acid metabolism and efferocytosis, etc.

### 3.5. Verification of the Interaction Between Host Proteins and CPV VP2 Capsid Protein

**Figure 5:**
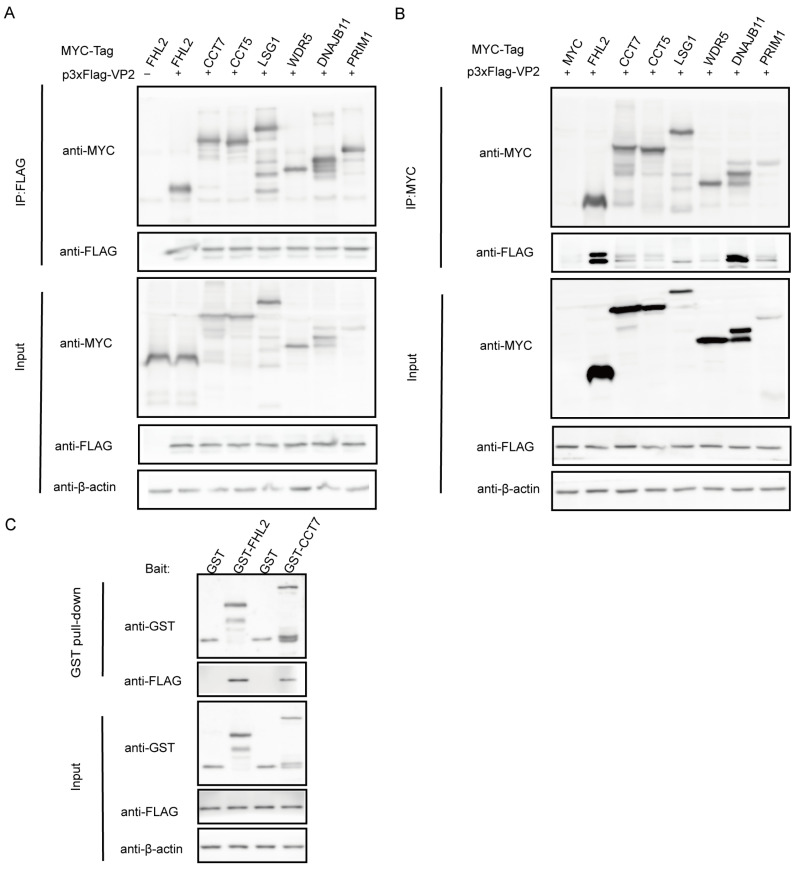
Validation of CPV VP2–host protein interactions. (**A**,**B**) HEK 293T cells were co-transfected with plasmids expressing MYC-FHL2, MYC-CCT7, MYC-CCT5, MYC-LSG1, MYC-WDR5, MYC-DNAJB11, MYC-PRIM1, and plasmid expressing p3xFlag-VP2, respectively, in which p3xFlag-VP2 or MYC FHL2 was co-transfected with the empty vector as a negative control, respectively. Cell lysates were immunoprecipitated with anti-Flag or anti-MYC, separated by SDS-PAGE, Western blotted and detected with the corresponding antibodies, respectively, and β-actin was used as an internal control. The molecular weights of the interacting host proteins are approximately 32KD (FHL2), 59KD (CCT7), 56KD (CCT5), 71KD (LSG1), 36KD (WDR5), 41KD (DNAJB11) and 52KD (PRIM1). (**C**) Whole-cell lysates of p3xFlag-VP2 were added to GST, GST-FHL2, and GST-CCT7 for the GST pull-down assay, respectively; immunoblotting was then performed with antibodies against GST, and β-actin was used as an internal control.

To verify the protein interaction from LC-MS, we conducted a Co-IP experiment in vitro. We randomly selected seven host proteins interacting with CPV VP2. p3xFlag-VP2 or empty vector was co-transfected with MYC-FHL2, CCT7, CCT5, LSG1, WDR5, DNAJB11 and PRIM1into HEK293T cells, and then immunoprecipitated with anti-FLAG and anti-MYC microbeads (Figure 5A,B). The results showed that CPV VP2 specifically interacted with FHL2, CCT7, CCT5, LSG1, WDR5, DNAJB11and PRIM1 and the binding strength was different, while no signal was observed in the empty vector control.

To further confirm whether CPV VP2 directly interacts with host proteins, we selected two host proteins for GST pull-down experiments. The lysis products of p3xFlag-VP2, GST-FHL2 and GST-CCT7 were tested by GST pull-down and Western blotting. As shown in Figure 5C, the results showed that CPV VP2 directly binds to FHL2 and CCT7. In summary, the results of Co-IP and GST pull-down experiments verified the data of LC-MS-based proteomics analysis.

### 3.6. FHL2 Inhibits Viral Replication

**Figure 6:**
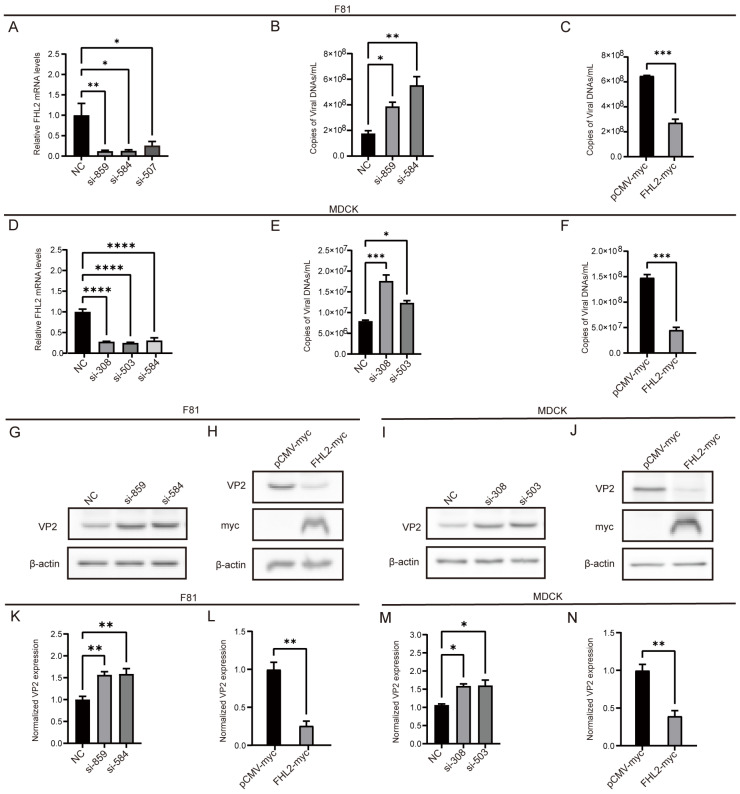
FHL2 inhibits CPV replication. (**A**,**D**) The mRNA levels of FHL2 in F81 and MDCK after si-NC or si-FHL2 treatment. The cells were transfected with si-FHL2 for 48 h, and the total RNA was extracted. The mRNA expression level of FHL2 in the cells was determined by fluorescence quantitative PCR. (**B**,**E**) After knocking down FHL2 in F81 and MDCK cells, the cells were infected with 0.1 MOI CPV, and the mRNA level of VP2 was evaluated by qPCR 48 h after infection. (**G**,**I**,**K**,**M**) The samples were as described in (**B**). The protein level of VP2 was determined by WB 48 h after infection, and the relative expression of VP2 in the samples was analyzed by Image J software. (**C**,**F**) F81 and MDCK cells were cultured in a 6-well plate. When cells grew to 70% to 90% on the plate, the empty vector (2 μg/well) or myc-FHL2 (2 μg/well) was transfected into the cells. After 24 h of transfection, the cells were infected with CPV at 0.1 MOI, and the mRNA level of viral VP2 was assessed by qPCR 48h after infection. (**H**,**J**,**L**,**N**) The sample was as described in (**C**). The protein level of virus VP2 was determined by WB 48 h after infection, and the relative expression of VP2 in the sample was analyzed by Image J software (Version 1.53). * *p* < 0.05, ** *p* < 0.01, *** *p* < 0.005 and **** *p* < 0.001 were considered statistically significant.

To evaluate the effect of FHL2 on CPV infection, siRNA was used to down-regulate the expression of FHL2 in F81 and MDCK cells. The qPCR results showed that the FHL2 gene was significantly down-regulated after interference (Figure 6A,D). Subsequently, cells were inoculated with 0.1 MOI virus, and viral replication was monitored by qPCR and WB-determined expression levels of CPV VP2 48h after infection. The results showed that the expression of CPV VP2 was significantly increased in FHL2 knockdown cells compared to control cells (Figure 6B,E,G,I,K,M). To evaluate the effect of FHL2 on CPV proliferation, we overexpressed it in F81 and MDCK cells. Western blot (WB) analysis confirmed the overexpression of FHL2 in cells (Figure 6H,J). Then, the cells were infected with 0.1 MOI for 48 h, and the expression level of CPV VP2 was evaluated by qPCR and WB (Figure 6C,F,H,J,L,N). The results showed that the overexpression of FHL2 resulted in a decrease in the expression of CPV VP2 protein. To sum up, these results confirm that FHL2 mediates CPV replication.

### 3.7. FHL2 Inhibits CPV Replication by Promoting IFN Transcription

**Figure 7:**
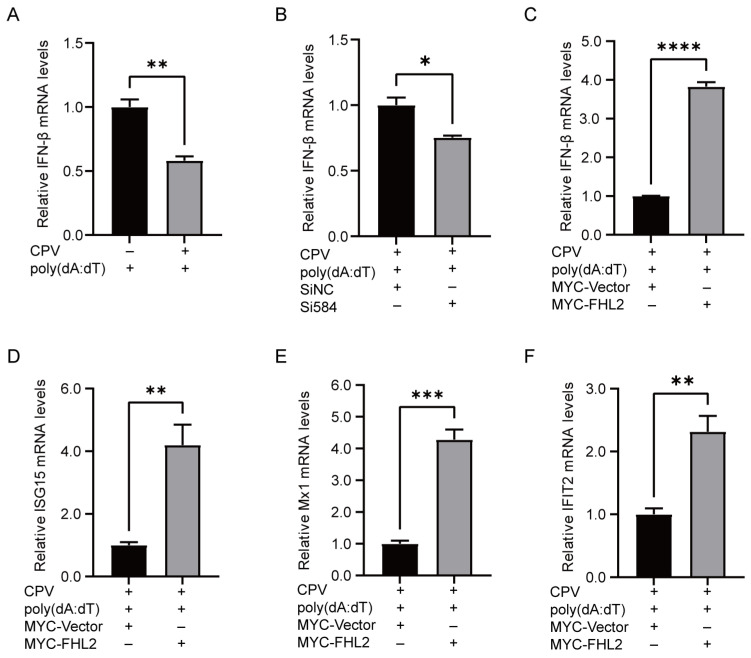
FHL2 can enhance innate immunity. (**A**) F81 was cultured in a 6-well plate, then infected with 0.1 MOI CPV, transfected with poly(dA:dT) (0.5 μg/mL) for stimulation 24 h later, and the mRNA level of IFN-β was measured by qPCR after 6 h. (**B**) In F81 cells with FHL2 knockdown, the cells were infected with 0.1 MOI CPV, and then transfected with poly(dA:dT) (0.5 μg/mL) for stimulation 24 h later. After 6 h, the cells were collected and the mRNA levels of IFN-β were recorded (**C**–**F**). In F81 cells with FHL2 overexpression, the cells were treated with 0.1 MOI CPV. Twenty-four hours later, the cells were transfected with poly(dA:dT) (0.5 μg/mL) for stimulation. After six hours, the mRNA levels of IFN-β, ISG15, Mx1 and IFIT2 were measured by qPCR. * *p* < 0.05, ** *p* < 0.01, *** *p* < 0.005 and **** *p* < 0.001 were considered statistically significant.

It has been shown that FHL2 inhibits SARS-CoV-2 replication by promoting IFN transcription [17]. We examined the expression level of IFN-β in F81 cells treated with poly(dA:dT) and infected with CPV by fluorescence quantitative PCR. As Figure 7A shows, CPV infection significantly reduced IFN-β transcription. Subsequently, we tried to confirm whether FHL2 has a similar regulatory effect on IFN-β in F81 cells. We detected the transcription level of IFN-β in F81 cells infected with CPV under the condition of FHL2 overexpression or knockdown (Figure 7B,C). In addition, we examined the effect of FHL2 on the transcription of downstream interferon-stimulated genes (ISGs), including ISG15, Mx1 and IFIT2. The pCMV-myc-FHL2 was transfected into F81 cells, infected with CPV, and then stimulated with poly(dA:dT). The mRNA levels of the above genes were detected by qRT-PCR (Figure 7D–F). The results showed that overexpression of FHL2 significantly increased the mRNA transcription level of ISGs. The above results demonstrate that FHL2 limits CPV replication by enhancing innate immunity.

## 4. Discussion

Parvovirus is one of the smallest viruses. Multiple deep-sequencing studies have shown that “fossil” parvovirus sequences have been integrated into the genomes of many animals, including humans [18]. Due to their very high level of host adaptation, most animal species are infected with at least one parvovirus, and usually more viruses [19]. Many of these viruses produce little or no disease, but canine parvovirus (CPV) can cause fatal disease in susceptible hosts. Currently, vaccination is the most effective measure to control virus transmission in dogs and prevent clinical CPV infection [20], but there is no specific antiviral treatment for CPV-2, and the main approach is symptom-based supportive care [21]. The canine parvovirus capsid protein has highly complex interactions with its host; elucidating these relationships and their roles is essential for a better understanding of the complexity behind the ability of canine parvovirus to jump from one host to another and to effectively maintain transmission and viral replication, and can lay the foundation for the development of vaccines and antiviral drugs

In this study, we first identified the host protein interacting with CPV VP2 protein in F81 cells by Co-IP pull-down combined with LC-MS/MS technique, and mapped the PPI network. Analysis of the results showed that host proteins targeted by CPV VP2 proteins mainly formed a PPI network map centered on the endoplasmic reticulum and ribosomes. It is well known that viral-encoded proteins are synthesized on the endoplasmic reticulum and then transported to the cytoplasm for folding and assembly, which plays an important role in the assembly and spread of the virus [22,23].

Notably, the 140 CPV VP2 potential interacting proteins identified include the TRIC/CCT molecular chaperone proteins CCT2, CCT7, CCT5, and CCT8. These proteins have been found to be involved in the regulation of protein processing, genome replication and viral assembly [24,25,26,27]. Our research group previously demonstrated the interaction between TRIC/CCT chaperone CCT7 and CPV VP2 protein through yeast two-hybrid experiments. Further studies have found that CCT7 promotes viral replication by improving the stability of VP2 protein [15]. In view of the potential role of TRIC/CCT molecular chaperone proteins in viral proliferation, whether CCT2, CCT5, or CCT8, mediate the proliferation of CPV, and its specific molecular mechanism still needs further study.

GO analysis results show that host proteins interacting with CPV VP2 protein are mainly distributed in ribosomes and endoplasmic reticulum, so CPV VP2 protein mainly targets the transport and assembly of viral proteins. The results of KEGG show that host proteins interacting with CPV VP2 are mainly involved in DNA replication, endoplasmic reticulum protein processing, Ribosome biogenesis in eukaryotes, AMPK signaling pathway and other processes. Studies have found that the AMPK signaling pathway is related to autophagy and apoptosis [28]. Therefore, CPV VP2 may regulate autophagy and apoptosis by targeting key proteins in the AMPK signaling pathway. However, the specific molecular mechanism still needs further study.

In this study, we randomly selected seven host proteins, including FHL2, CCT7, CCT5, LSG1, WDR5, DNAJB11and PRIM1 and demonstrated their interaction with CPV VP2 protein through Co-IP experiments. In addition, the direct interaction between CCT7, FHL2 and CPV VP2 was further confirmed through GST pull-down experiments. Among them, we preliminarily explored the role of FHL2 in viral replication. The four-and-a-half LIM structural domain 2 (FHL2) belongs to the LIM-only family of proteins, which mainly serve as scaffolding proteins and articulators to support the assembly of multimeric protein complexes [29]. We observed that overexpression of FHL2 in F81 and MDCK cells would inhibit virus replication, while knocking down FHL2 gene would promote virus replication. Previous studies have shown that FHL2 is a key regulator of immune response in virus infection, and it can regulate IFN-β to inhibit virus replication [17,30,31]. The role of type I interferons in the replication of porcine parvovirus has been well elucidated [32]. Studies on other viruses, such as SARS-CoV-2, have shown that FHL2 enhances the production of interferons by promoting the expression, nuclear translocation, and phosphorylation of IRF3. In addition, FHL2 interacts with TRAF2 and TRAF6 to regulate NF-κB activity. In this study, we found that FHL2 enhances the transcription of IFN-β and inhibits the replication of CPV [17,33]. However, whether FHL2 regulates interferon production through the aforementioned pathways and subsequently mediates CPV replication remains to be further investigated. A deeper understanding of the mechanisms of FHL2 will provide a theoretical foundation for the development of future antiviral therapies.

## 5. Conclusions

In a word, we identified host proteins interacting with CPV VP2 in F81 cells for the first time. Through analysis, we found that these proteins are mainly involved in cell metabolism, cell biosynthesis, protein folding and various signal transduction pathways. After that, seven host proteins were randomly selected for Co-IP and/or GST pull-down experiments, and the experimental results were consistent with those of mass spectrometry. Furthermore, our subsequent study indicated that modulation of FHL2 gene expression impacts viral replication, and the activation of the interferon pathway may be involved in this process. Our study further confirms the interaction between the structural proteins of CPV and host proteins, providing a solid scientific foundation for the future development of antiviral agents and vaccines targeting viral replication and immune evasion mechanisms.

## Figures and Tables

**Table 1:** List of primers used for cloning in the study.

Gene	Sequence 5′-3′
Flag-VP2-F*	GCGAATTCCATGAGTGATGGAGCAGTTCAA
Flag-VP2-R*	CTGGTACCGTGTATAATTTTCTAGGTGCTA
Myc-FHL2-F	CGGAATTCATGATGATGACGGAGCGCTTTGACT
Myc-FHL2-R	CCGCTCGAGGGATGTCTTTCCCACAGTCCGGG
Myc-CCT5-F	CGGAATTCATGGCAACGGAGGAGAAGAAGC
Myc-CCT5-R	CCGCTCGAGGTTCTTCGGATTCCCCAGGCT
Myc-CCT7-F	CCGAATTCACATGATGCCCACACCAGTTAT
Myc-CCT7-R	ACCTCGAGGGTGGGGGCGGCCCCGGCCCCG
Myc-LSG1-F	CGGAATTCATGGGCCGTAGAAGAGCGC
Myc-LSG1-R	CCGCTCGAGGCGTATCCAGGTGCTTATAGAGTCTACGAG
Myc-WDR5-F	CGGAATTCATGGCAACGGAGGAGAAGAAGC
Myc-WDR5-R	GGGGTACCGCAGTCACTCTTCCAGAGCTTAATGG
Myc-DNAJB11-F	CGGAATTCATGGCCCCGCAGAACCT
Myc-DNAJB11-R	GGGGTACCATATCCTTGCAGTCCATTGTATACCTTCTGC
Myc-PRIM1-F	CGGTCGACATGGAGACGTTTGACCCCGCA
Myc-PRIM1-R	CGGGTACCGAAGTCTTTCTGTAAATCACTCTTCTTGAGAAGT
GST-FHL2-F	CGGAATTCATGATGATGACGGAGCGCTTTGACT
GST-FHL2-R	CCGCTCGAGTCAGATGTCTTTCCCACAGTCCGGG
GST-CCT7-F	CGGAATTCATGATGCCCACACCAGTTATCCTG
GST-CCT7-R	CCGCTCGAGTCAGTGGGGGCGGCC

F* denotes forward PCR primer; R* denotes reverse PCR primer.

**Table 2:** List of primers used for RNA interference.

Gene	Sense (5′-3′)	Antisense (5′-3′)
FHL2-fca-507	GAUGGAGUACAAGGACAGCTT	GCUGUCCUUGUACUCCAUCTT
FHL2-fca584	GCUUCAUCCCUAAGGACAATT	UUGUCCUUAGGGAUGAAGCTT
FHL2-fca-859	GGUGGCACAAAGUACAUCUTT	AGAUGUACUUUGUGCCACCTT
FHL2-cfa-308	GCUGUGACUGCAAGGACUUTT	AAGUCCUUGCAGUCACAGCTT
FHL2-cfa-503	GCAAGAUGGAGUACAAAGATT	UCUUUGUACUCCAUCUUGCTT
FHL2-cfa-584	GCUUCAUCCCUAAGGACAATT	UUGUCCUUAGGGAUGAAGCTT

**Table 3:** List of primers used for qPCR.

Gene	Sequence 5′-3′	Species
VP2-F*	CAAATAGAGCATTGGGCTTACC	
VP2-R*	TCCCATTTGAGTTACACCACG	
β-actin-F	CATGTACGTGGCCATCCAGGC	Felis Catus
β-actin-R	CTCCTTGATGTCACGCACAAT	Felis Catus
IFN-β-F	CAAATCGCTCTCTTGGTGT	Felis Catus
IFN-β-R	AAACTGCTGCTTCTTAGTTGG	Felis Catus
FHL2-F	CTGCACCAACCCCATCAGC	Felis Catus
FHL2-R	TGAAACAGTCGTTGTGCCACT	Felis Catus
ISG15-F	TGCCCCTGAGAGACAACATGC	Felis Catus
ISG15-R	GAACCCCATCGCGCAGCAC	Felis Catus
Mx1-F	CAAGAAGAACCTATGTAGCCAGT	Felis Catus
Mx1-R	AGCTCTGGTCTCCGATGACA	Felis Catus
IFIT2-F	GCAGCCCCTACAGAATCGAG	Felis Catus
IFIT2-R	GCCTTCTCAAAGCACACCT	Felis Catus
FHL2-F	CTCCAAGTGCCAGGAATGCAA	Canis lupus familiaris
FHL2-R	ATGAAGCTCTTGGTACCGAT	Canis lupus familiaris

F* denotes forward PCR primer; R* denotes reverse PCR primer.

## Data Availability

The original contributions presented in this study are included in the article/Supplementary Material. Further inquiries can be directed to the corresponding authors.

## Supplementary Materials

The following supporting information can be downloaded at: https://www.mdpi.com/article/10.3390/microorganisms13010088/s1, Figure S1: The GO analysis of CPV VP2-interacting host proteins using the GOclue plug-in in Cytoscape software version 3.10.1, *p* < 0.05; Table S1: Host proteins interacting with FLAG-VP2 in F81 cells transfected with p3xFlag-CMV (708) and p3xFlag-VP2 (763); Table S2: The GO annotation analyses of CPV VP2-interacting host proteins; Table S3: The KEGG enrichment analyses of CPV VP2-interacting host proteins.
