# Supplementary figures and images for: Identification of Host–Protein Interaction Network of Canine Parvovirus Capsid Protein VP2 in F81 Cells
Source: Microorganisms. 2025 Jan 5;13(1):88. doi: 10.3390/microorganisms13010088 (PMC11767315; doi:10.3390/microorganisms13010088)

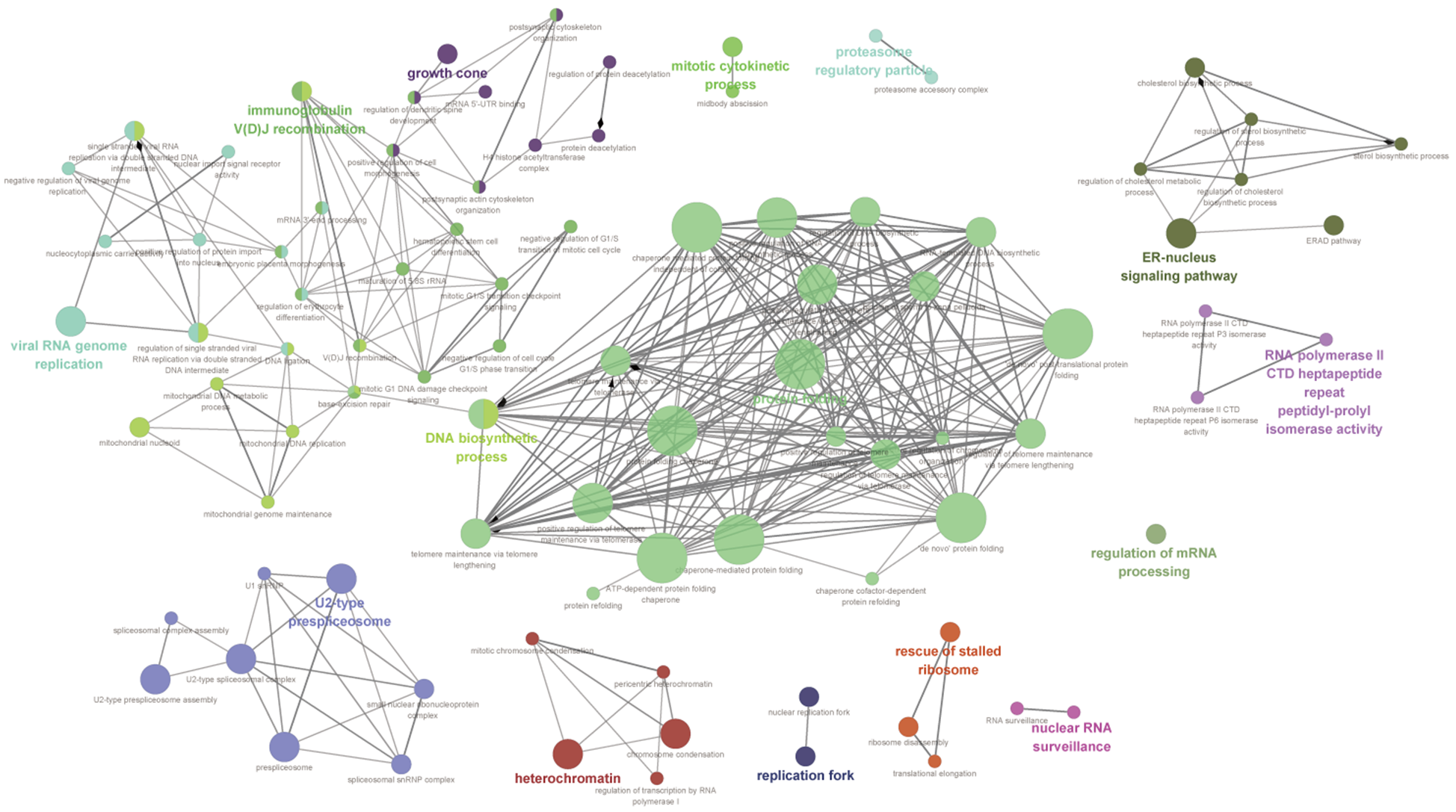

Supplement: Supplementary file 1 [file microorganisms-13-00088-s001.zip › Supplementary Figure S1.TIF]
